# Survey of public data sources on the Internet usage and other Internet statistics

**DOI:** 10.1016/j.dib.2018.04.107

**Published:** 2018-05-09

**Authors:** Murooj Nadhom, Pavel Loskot

**Affiliations:** College of Engineering, Swansea University, Swansea SA1 8EN, United Kingdom

## Abstract

The Internet research is mainly driven by data. Obtaining such data by planning and launching measurement campaigns is rather time consuming and costly. Much more efficient, and in many cases, sufficient data acquisition strategy is to exploit the existing datasets available in public databases, repositories, and documents. Hence, the public data sources related to the Internet usage and other Internet statistics are systematically surveyed and categorized to make the search for the Internet data much easier and faster. Extensive online searches and exploring websites of the key organizations were used to identify the data sources. Each data source was then carefully explored to describe its characteristics and contents. The data are usually aggregated over certain time periods and regions, and often indexed by age, gender, application, website, activity and other attributes. Some data sources also support various data visualization options, and offer data export in multiple formats.

**Specifications Table**

**Table:** 

Subject area	*Engineering, Computer Science*
More specific subject area	*Computer networks, Telecommunications*
Type of data	*5 Table, 1 Figure*
How data was acquired	*Survey of public datasets on WWW*
Data format	*URL with brief description*
Experimental factors	*Each dataset was explored for its contents and then categorized.*
Experimental features	*Guided searches on WWW. Search of WWW of relevant organizations.*
Data source location	*N/A*
Data accessibility	*Included with this data brief.*

**Value of the data**

• There is no other centralized collection of public datasets on the Internet usage and other Internet statistics while searching for such datasets is a very time consuming process.
• The identified data sources can be used to identify and understand the current and future trends in the Internet usage.
• Having the Internet usage data and statistics is important to understand a rapidly ongoing digitalization of many sectors of the economy including manufacturing, healthcare, education and entertainment.
• It is vital that data sources on the Internet usage are regularly updated and made available to the researchers as well as policy and decision makers in order to keep evolving the Internet for the maximum benefits of the society.

## 1 Data

There are different types of data associated with the Internet. The Internet measurements are used for real-time network management, long-term network planning as well as to evaluate how the Internet is used and its impact on the economy and the society. The measurements used for network management are obtained directly from the Internet traffic flows. The network planning for 1 or 2 years ahead of the current demand is based on the traffic trend forecasting by extrapolating historical data. For longer-term planning beyond a 2 year horizon and to evaluate the long-term and large scale impacts of the Internet, the user questionnaires and other market research methods are necessary. The Internet data are regularly published by the government institutions, regulatory bodies, communication service providers, and private organizations. The data with low level of aggregation are usually only accessible for a fee. The published Internet usage data are highly aggregated which reduces the resolution, but also the variations in data. In order to obtain measurements for high volume and high throughput traffic, or to efficiently represent the Internet topology at multiple time scales, statistical sampling strategies are required to process smaller but statistically relevant traffic traces.

The collected datasets were somewhat liberally organized in the following 5 tables:

Table 1. Internet measurements: These datasets mostly contain data on the Internet usage in a given region such as the number of subscribers, cellular coverage, security issues and the quality of service including access speeds and response times.

**Table 1:** Internet measurements.

	**Data source**	**Data**	**Notes**
1.	Individual usage of the Internet.	1960–2015: Number of Internet users. Fixed broadband subscriptions. Fixed telephone subscriptions. Secure Internet. servers. Mobile cell. subscriptions.	Yearly updates. XLS, XML and CSV format. Indexing by global, region, country and income. In 5 langs.^b^
	The World Bank Group.		
	http://data.worldbank.org		
2.	The World Factbook.	2014: Number of Internet users.	Updated 2014 (15th ed.). TXT format. Indexing by country. In 8 langs.
	The CIA.		
	https://www.cia.gov		
3.	ICT facts and figures.	2000 and 2015: Internet users.	A report published in 2015. PDF format. Indexing by global, region, country and level of development.
	ITU.	2011 and 2015: 3G mobile broadband coverage.	
	https://www.itu.int		
		2005–2015: Mobile and fixed broadband subscriptions. Individuals using Internet. Household Internet access. 2008–2014: Internet prices.	
		2014: Internet speeds.	
4.	ICT Fact and figures.	2005–2014: Fixed and mobile broadband subscriptions. Individuals using Internet. Household Internet access 2013: Internet speeds.	A report published in 2014. PDF format. Indexed by country and level of development.
	ITU.		
	https://www.itu.int		
5.	Measuring the information society.	2020: The targets.	A report published in 2015. PDF format. Indexed by global, level of development, gender, region and country.
	ITU.	2015: Internet access.	
	https://www.itu.int	2005–2015: Individuals using the Internet. 2005–2015: Household Internet access. 3G network coverage.	
		2014: Cyber security level.	
		2010 and 2015: Internet development induction.	
		2008–2014: Internet prices and speeds.	
		2000–2015: Fixed and mobile broadband subscriptions. Individuals using Internet. Household Internet access.	
6.	Women's rights online.	2014: Internet user and access. Barriers to using Internet. Reasons for using social media. Internet use in political and economic issues.	A report published in 2015. PDF format. Indexed by gender, education, age and gender. For 9 countries.
	WWW Foundation.		
	http://webfoundation.org		
7.	Men vs. Women: Who Is more active on social media?	2014: Internet user activity in social media.	A 2015 online article. PNG and BMP format. Indexed by gender. In 5 langs.
	Iris Vermere.		
	https://www.brandwatch.com		
8.	Map of Internet users.	2014: Internet users.	Last update in 2012. PNG format. Indexed by country and gender.^a^^,^^b^
	Visually website.		
	https://visual.ly		
9.	Internet users by country and gender.	2000–2014: Internet users.	Last update in 2014. JPG and TXT format. Indexed by level of development, country and gender.^a^
	Gilly Wright.		
	https://www.gfmag.com		
10.	Global Internet report.	2007–2012: Internet users.	Survey published in 2014. PDF format. Indexed by global, region and gender. For 20 countries.
	Internet Society Organization.	2008–2012: Fixed Internet subscriptions. Mobile broadband.	
	http://www.internetsociety.org	2010–2018: Fixed Internet traffic.	
		2011–2018: Mobile Internet traffic.	
		2013: Traffic generated different apps. 2014: Open and sustainable Internet survey.	
11.	UNECE Statistical Database. UNECE.	1990–2016: Internet use	Yearly, quarterly and weekly updates. XLS, CSV and PX format. Indexed by age, gender and country.^a^^,^^b^
	http://w3.unece.org		
12.	Over a quarter of the world's Internet users are aged 16–24.	Q3-Q4 2014: Internet users.	Article published in 2015. PNG and BMP format. Indexed by age (16–24), global and selected country.^a^
	Global Web Index by Jason Mander.		
	http://blog.globalwebindex.net		
13.	World Internet users.	1960–2015: Internet users.	Yearly updates. XML and CSV format. Indexed by global, region and country.^a^
	UN Data Organization.		
	http://data.un.org		
14.	Internet users - for all countries.	1990–2011: Internet users.	Last update in 2011. SVG, PNG and BMP format. Indexed by country. In 3 langs.^a^^,^^b^
	Fact Fish.		
	http://www.factfish.com		
15.	Internet users - country rankings.	1960–2015: Internet users.	Yearly. Free for JPG. Not free for Excel and CSV format. Indexed by global, region and country. In 6 langs.^a^
	The Global Economy.		
	http://www.theglobaleconomy.com		
16.	Internet use and income.	2007–2014: Internet use and income.	SVG, PNG and BMP format. Indexed by country.^a^
	Internet Monitor.		
	https://dashboard.thenetmonitor.org		
17.	Real time web monitor for internet traffic and attack.	Real time web monitor for Internet traffic and attack.	Continuous updates. SVG,
			PNG and BMP format. Indexed by country. In 8 langs.
	Akami.		
	https://www.akamai.com		
18.	Traffic data.	Traffic index, response times and packet losses.	Daily, weekly and monthly updates. GIF and BMP format. Indexed by region and country.
	Internet Traffic Report.		
	http://www.internetttrafficreport.com		
19.	Fixed broadband subscriptions.	Internet users.	Yearly updates. Indexed by region and country.^a^
	Index Mundi.		
	http://www.indexmundi.com		
20.	Distribution of Internet users in Europe.	2014: Internet users in Europe.	Last update 2014. PNG, XLS, PPT and PDF format. Indexed by age. Sourced from more than 18,000 datasets. In 4 langs.
	Statista.		
	https://www.statista.com		
21.	Internet users.	2008–2010: Internet users.	Last update in 2012. JPG and BMP format. Indexed by gender and country in Europe.
	ITU.		
	http://www.itu.int		
22.	Internet users.	1990–2015: Internet users.	Yearly updates. XLS, PDF, PPT, SVG, PNG, BMP, CSV and TSV format. Indexed by income and region.^a^
	Data Market.		
	https://datamarket.com		
23.	Internet users.	1990–2015: Internet users.	Yearly updates. XLS, PDF, PPT, JPG and CSV format. Indexed by global and country.^a^
	FRED.		
	https://fred.stlouisfed.org		
24.	Middle East Internet users to hit 413 million in 2015.	2015: prediction of	Article published in 2013. JPG and BMP format.
	Arabian Gazette Ayesha Sarfraz.	Internet users in the Middle East.	
	https://arabiangazette.com		
25.	Top 10 Internet countries in Africa.	2014: Most connected countries in Africa.	Article published in 2014. PNG and BMP format. Indexed by users and country.^a^
	IT Web Africa by Gareth van Zyl.		
	http://www.itwebafrica.com		
26.	Africa's top 20 most connected countries.	2015: Top 20 connected countries in Africa.	Article published in 2015. TXT format. Indexed by users and country.^a^
	IT News Africa.		
	http://www.itnewsafrica.com		
27.	Africa Internet User Statistics.	2000 and 2015: Internet users in Africa.	Data published in 2016. TXT format. Indexed by country.^a^
	Statistic Brain.		
	http://www.statisticbrain.com		
28.	Latin American Internet Audience Nears 310 Million.	2013–2018: Internet users in Latin America.	Article published in 2014. GIF and BMP format. Indexed by country.
	eMarketer.		
	https://www.emarketer.com		
29.	Internet User in the UK.	2011–2017: Internet users in the UK.	Yearly updates. XLS format. Indexed by age, gender, economy and location.^a^
	ONS.		
	https://www.ons.gov.uk		
30.	Internet Users in Georgia.	2012: Internet users in Georgia.	Data published in 2013. PNG and BMP format. Indexed by regions, cities, operators and activities.^a^
	IDFI.		
	https://idfi.ge		
31.	India to have the second-largest internet user base in the world.	2015: Internet users in India.	Article published in 2015. PNG and BMP format. Indexed by rural and urban area.
	Your Story by Radhika P Nair.		
	https://yourstory.com		
32.	Internet Users in the USA.	2013: Internet users in the USA.	Yearly updates. XLS format. Indexed by gender, race, age, education and income.^a^
	NCES. U.S. Dept. of Education.		
	https://nces.ed.gov		
33.	First Look: Internet Use in 2015.	2013–2015: Internet users in the USA.	Report published in 2016. PNG and BMP format. Indexed by age and education level.^a^
	NTIA.		
	John B. Morris.		
	https://www.ntia.doc.gov		
34.	United Kingdom fact file.	2011–2016: Internet users.	Yearly updates. TXT format. Indexed by country.^a^ Some data not free.
	Euro Monitor.		
	http://www.euromonitor.com		
35.	Share of the Internet users.	2000–2015: Internet users.	Yearly updates. XLS, PPT, PDF, SVG, PNG and BMP format. Indexed by country. In 10 langs.^a^^,^^b^
	Knoema.		
	https://knoema.com		
36.	Internet use in the Middle East and North Africa.	2012: Internet use in Middle East and North Africa.	Article published in 2013. JIF and BMP format. Indexed by region.
	eMarketer.		
	https://www.emarketer.com		
37.	A snapshot of Internet use in Africa.	2012: Internet use in Africa.	Article published in 2013. JIF and BMP format. Indexed by region.^a^
	PC Mag. by Stephanie Mlot.		
	http://uk.pcmag.com		
38.	Internet users.	2017: Internet users.	Yearly updates. TXT, PNG and BMP format. Indexed by region.^a^
	Internet World Stats.		
	http://www.internetworldstats.com		
39.	Internet topology data.	2017: Macroscopic Internet topology data kit.	Router level topology data on the Internet nodes and links.
	CAIDA.		
	http://www.caida.org/data/		
40.	Internet topology data.	2017: University of Adelaide project of Internet topologies.	250 datasets on various Internet networks at different scales.
	Topology Zoo project.		
	http://topology-zoo.org		

a Other data available.

b Various visualization options available.
Table 2. Internet applications: These datasets can be used to evaluate how the users spent their time on the Internet, often focusing on the specific regions of the world and specific websites such as social networking applications or Youtube.

**Table 2:** Internet applications.

	**Data source**	**Data**	**Notes**
1.	World internet and digital future.	2009–2015 and 2000–2016: Internet usage in social, political, media, entertainment, security and economy for selected countries.	Project reports. PDF format. Indexed by age, gender, income, location and education.
	Digital Centre Organization		
	http://www.digitalcenter.org		
2.	Global internet usage.	2013: Global internet usage.	A PDF report. Indexed by region, language, browsers, search engine, social media and mobile vs. desktop.
	Build Export.		
	https://build.export.gov		
3.	Daily time spent on social networks rises to over 2 h.	2012–2016: Time spent on social networks.	Article published in 2017. PNG and BMP format. Indexed by avg. daily time.^a^
	Global Web Index Jason Mander.		
	http://blog.globalwebindex.net		
4.	How people spent time on the Internet.	2012: How users spent time on the Internet.	Infographics published in 2012. GIF and BMP format. Indexed by global, activity, website and apps.^a^
	Go-Gulf.		
	https://www.go-gulf.com		
5.	Internet access and use in Europe.	2006–2008: Internet access and use in Europe.	Data published in 2008. PDF and DOC format. Indexed by country.^a^
	European Commission.		
	https://ec.europa.eu		
6.	Internet usage in Asia-Pacific.	2000–2010: Internet usage in Asia-Pacific.	Infographic. JPG and BMP format. Indexed by selected countries.
	Visually.		
	https://visual.ly		
7.	Internet usage in Asia-Pacific.	2007: Internet usage in Asia-Pacific.	Data published in 2007. TXT format. Indexed by global, countries, region and avg. time spent on website.^a^
	comScore Inc.		
	http://www.comscore.com		
8.	Asia Internet Usage.	2000 and 2010: Asia Internet Usage.	Data published in 2010. PNG and BMP format. Indexed by selected countries.
	SCRIBD.		
	https://www.scribd.com		
9.	Mobile Internet Usage in Asia-Pacific.	2016: Mobile Internet usage in Asia-Pacific.	Report published in 2016. PDF format. Indexed by activity, operator, device, age and app.
	Internet Society Organization.		
	https://www.internetsociety.org		
10.	The Arab World Online 2014.	2014: Arab world online.	Report. PDF format. Indexed by device, location, spending time, social media, age, income, and app.^a^
	Mohammed Bin Rashid School of Government.		
	http://www.mbrsg.ae		
11.	Internet usage in the Middle East.	2001, 2012 and 2015: Internet usage in the Middle East.	Infographics published in 2013. Indexed by country and app.^a^
	Go-Gulf.		
	https://www.go-gulf.ae		
12.	Internet usage in the Middle East.	Internet Usage in the Middle East.	Infographics. JPG and BMP format. Indexed by access and app.
	Info Graphicspedia http://www.infographicspedia.com/author/admin/		
	http://www.infographicspedia.com		
13.	Mobile internet usage in the Middle East.	2014–2017: Mobile internet usage in the Middle East.	Infographics. PNG format. Indexed by age, device, users, employment, education, gender and app.
	https://www.middleeast-infographics.com		
14.	Out-of-home use of the Internet.	2014: Internet use in the UK	Report published in 2014. PDF format. Indexed by activity, downloading speed, device, age and app.
	BSG.		
	http://www.broadbanduk.org		
	Ofcom.	2011–2017: Internet use in the UK.	Reports. PDF format. Indexed by location, device, activities, game, browsing, access, age and app.^a^
	https://www.ofcom.org.uk	Adults media use and attitudes.	
		2013: Internet use in the UK.	Reports. PDF format. Indexed by gender, age, activities and use of websites.^a^
		Internet citizens.	
		2016: Internet use in the UK.	Reports. PDF format. Indexed by speed and operator.^a^
		Smartphone cities.	
		2014 and 2015: Mobile broadband performance in the UK.	Reports. PDF format. Indexed by operator, speed, latency, cities, device, activity, speed, and operator.^a^
		2011–2016: Internet use and attitudes in the UK bulletin.	Reports. PDF format. Indexed by age, gender, income, unemployed, rural/urban area, ethnicity and disability.^a^
15.	Internet and computer use in London.	2013: Internet and computer use in London area.	Data published in 2013. XLS format. Indexed by access.^a^
	Datahub.		
	https://datahub.io		
16.	Mobile connectivity research study.	2016: Internet use in the UK trains.	Report published in 2016. PDF format. Indexed by device, spend time, connectivity and activity.
	UK Government.		
	https://www.gov.uk		
17.	How internet use has ballooned in the last decade.	2012–2014: Internet use among teenagers in the UK.	Article published in 2015. SVG, PNG, BMP, JPG and PDF format. Indexed by device and social website.
	The Telegraph.		
	http://www.telegraph.co.uk		
18.	India Internet Usage.	2015: Internet usage in India.	Infographic. PDF format. Indexed by speed, time spend, gender, area and speed.^a^
	Google.		
	https://storage.googleapis.com		
19.	Internet Usage in China.	2012: Internet usage in China.	Infographic published in 2013. Indexed by device, gender, area, access and activity.^a^
	Go-Globe.		
	https://www.go-globe.com		
20.	Internet Usage in China.	2013: Internet Usage in China.	Infographics. PNG and BMP format. Indexed by gender, age, education, occupation, device and activity.^a^
	Simply Mandarin.		
	http://simplymandarin.com		
21.	Internet usage in Hong Kong.	2013 and 2014: Internet usage in Hong Kong.	Infographics published in 2014. Indexed by device, access, activity, age, gender, spent time and area.^a^
	Go-Globe.		
	https://www.go-globe.hk		
22.	Internet use in Canada.	2005, 2007 and 2009:	Last update 2010. XLS, CSV and HTML format. Indexed by individuals, location of access and province.^a^
	Statistics Canada.	Internet use in Canada.	
	http://www.statcan.gc.ca		
23.	Internet usage in Brazil.	2014: Internet usage in Brazil.	Last update 2014. TXT format. Indexed by activity, access, spend time and app.
	Tech in Brazil.		
	https://techinbrazil.com		
24.	Internet usage in Brazil.	2015: Internet usage in Brazil.	Published in 2016. PNG and BMP format. Indexed by device, activity and app.
	Business of apps.		
	http://www.businessofapps.com		
25.	Global internet industry research.	Different years.	Report. PDF format. Indexed by operator, device, age, activity, social media and app.
	Avazu.		
	http://avazuinc.com		
26.	The 100 websites that rule the internet in the USA.	2017: Top 100 websites in the USA.	Infographic published in 2017. JPG format.
	Visual Capitalist. http://www.visualcapitalist.com		
27.	The state of the Internet.	State of the Internet connectivity, security and speeds.	Quarterly updates. Data form 2008. Indexed by region and country. In 8 langs.
	Akamai		
	https://www.akamai.com/us/en/about/our-thinking/state-of-the-internet-report/https://www.akamai.com		
28.	The Internet usage.	The top 500 sites on the web.	Daily updates. Indexed by global, country, category, spent time, traffic, website and apps. Free and non-free data.
	Alexa		
	https://www.alexa.com		
29.	Measuring broadband America.	Lots of data and reports on telecommunications and the Internet in the US and international. Mobile and fixed broadband, QoS, Internet access, high-speed access.	Reports from as early as 1985 in PDF or TXT formats. Index by performance, coverage, subscriptions, pricing,
	FCC		
	https://www.fcc.gov		
30.	Social apps usage.	User data for YouTube, Twitch, Instagram and Twitter.	Rich indexing and filtering.
	Social Blade		
	https://socialblade.com		
31.	Personal data on Google.	Access to one's own personal data on Google.	Export, share and manage personal data on Google.
	Google account.		
	https://myaccount.google.com		
32.	Social apps usage.	Usage data for selected social applications including Youtube, Facebook and Twitter.	Indexed by country, global, industry, most active profiles, daily and monthly uses.
	Social Bakers		
	https://www.socialbakers.com		
33.	Wiki groups statistics.	Statistics about wiki groups.	Monthly update. Various formats.
	Wikimedia		
	https://stats.wikimedia.org/		
34.	Media use in the Middle East.	2016: Data about the Internet and social networks in the Middle East.	Report. PDF format. Indexed by country, penetration, device, usage, age and app.
	North Western University in Qater.		
	http://qatar.northwestern.edu		
35.	http://www.socialblade.com	Usage statistics on Youtube, Twitch, Instagram, Twitter and Daily motion.	HTTP tables with sorting. Also in real time.

a Other data available.
Table 3. Various Internet statistics: These datasets provides information about the Internet usage and usage analytics in various parts of the world.

**Table 3:** Various Internet statistics.

	**Data source**	**Data**	**Notes**
1.	The Internet statistics and resources.	2001–2016: a lot of various Internet statistics in the USA and worldwide.	Last updated in 2016. TXT format. Indexed by gender, age, race, income, education, employment, region and country.^a^
	Info Please.		
	http://www.infoplease.com		
2.	Smartphone Ownership and Internet Usage Continues to Climb in Emerging Economies.	2000–2017: Internet statistics.	Data published in 2017. BNG and BMP format. Indexed by country, age, education, income, access and devices.^a^
	Pew Research Center.		
	http://www.pewglobal.org		
3.	The Internet.	Internet statistics, various years.	BNG and BMP format. Indexed by country, access, economy and app.^a^^,^^b^
	Our World in Data.		
	https://ourworldindata.org		
4.	Social Media Facts and Statistics.	2013 and 2015: Social media statistics.	Articles. TXT format. Indexed by age, gender, region, device and app.^a^
	Jeff Bullas.		
	http://www.jeffbullas.com		
5.	Internet Stats & Facts for 2016.	2016: Internet statistics.	Article published in 2016. TXT, BNG and BMP format.^a^
	Hosting Facts.		
	https://hostingfacts.com		
6.	96 Amazing Social Media Statistics and Facts.	2016: Social media statistics.	Article published in 2016. TXT format. Indexed by app. In 5 langs.^a^
	Brand Watch.		
	https://www.brandwatch.com		
7.	Internet Trends 2017.	2017: Stats & Facts in the USA and Worldwide.	Article. JPG and BMP format. Indexed by region, users, age, device, speed, activity and browser. In 25 langs.^a^
	VPN Mentor Guy Fawkes.		
	https://www.vpnmentor.com		
8.	There are now 3 billion Internet users worldwide in 2015.	2015: Internet statistics in the UK.	Article published in 2015. PNG and BMP format. Indexed by use mobile web, mobile broadband, mobile data growth, spend time, speed, region, device, social media and app.^a^
	Mobile Industry Review.		
	http://www.mobileindustryreview.com		
9.	Mobile usage statistics.	Live mobile usage statistics.	Live updates. Indexed by activity, device and app.
	Internet Stats Today.		
	http://internetstatstoday.com		
10.	How the world uses the internet.	2009–2018: Internet statistics.	Infographics. PDF format. Indexed by region, country, activity. In 11 langs.^a^
	IDC.		
	http://www.idc.com		
11.	Internet trends.	2017: Global internet trends, advertisement, game, media, China and India internet and healthcare.	Report and presentation. JPG and BMP format.
	Kleiner Perkins Caufield and Byers.		
	http://www.kpcb.com		
12.	Internet use and statistics.	2013 statistics in Europe.	Article. PNG and BMP format. Indexed by users, age, activity, websites and shopping. In 22 langs.^a^
	Eurostat.		
	http://ec.europa.eu		
13.	Digital economy and society statistics - households and individuals.	2016 statistics in Europe.	Articles. PNG, BMP and XLS format. Indexed by access, usage and device. In 22 langs.^a^
	Eurostat.		
	http://ec.europa.eu		
14.	Social Media Report.	2015 and 2017: Social media statistics in Australia.	Reports. PDF format. Indexed by device, age, gender, access and social media.^a^
	Sensis.		
	https://www.sensis.com.au		
15.	Digital landscape of Southeast Asia.	2015: Internet statistics in Southeast Asia.	Presentation. JPG and BMP format. Indexed by Facebook usage, gender, age and country.^a^
	Tech in Asia.		
	https://www.techinasia.com		
16.	Digital landscape in Asia.	2015: Internet statistics in Asia.	Infographics published in 2015. Indexed by country, users, device, spend time and speed.^a^
	Go-Globe.		
	https://www.go-globe.com		
17.	Internet usage in Hong Kong.	2013 and 2014: Internet statistics in Hong Kong.	Infographics published in 2014. Indexed by device, access, activity, age, gender, spend time and area.^a^
	Go-Globe.		
	https://www.go-globe.hk		
18.	Internet user statistics in Asia.	2000 and 2016: Internet user statistics in Asia.	Data published in 2016. TXT format. Indexed by internet users, Facebook users and country.^a^
	Statistic Brain.		
	http://www.statisticbrain.com		
19.	Social media statistics in the Middle East.	2015: Social media statistics in the Middle East.	Annual survey. PNG and BMP format. Indexed by activity and app.
	Middle East media organization.		
	http://www.mideastmedia.org		
20.	Social media statistics in the Middle East.	2013: Social media statistics in the Middle East.	Infographics published in 2013. Indexed by language, age, gender, access and country.^a^
	Go -Gulf.		
	https://www.go-gulf.ae		
21.	Internet statistics in selected countries.	2015: Internet statistics in India.	Presentations. PDF and PPT format. Indexed by many different attributes.^a^
	Slideshare users.	2015: China mobile Internet statistics.	
	https://www.slideshare.net	2014: Internet statistics in Russia.	
22.	Global internet report in Latin America and North America.	2015: Internet statistics in America.	Report. PDF format. Indexed by access and activity.^a^
	Sandvine.		
	https://www.sandvine.com		
23.	Irish communications market.	2015: Internet statistics in Ireland.	Report. PDF format. Indexed by access, usage, penetration, pricing, users, operators, speed and traffic.
	Commission for Communications Regulation		
	https://www.comreg.ie		
24.	Analysis of broadband performance in Ireland.	2014: Broadband quality in Ireland.	Study. PDF format. Indexed by download, upload, latency and packet loss.
	Sam Knows.		
	https://www.samknows.com		
25.	Statistics of Internet Users in Georgia.	2012: Internet statistics in Georgia.	Data published in 2013. PNG and BMP format. Indexed by activity, region, operators and access.
	IDFI.		
	https://idfi.ge		
26.	Social Media Users in Argentina.	2015: Social media statistics in Argentina.	Article published in 2015. PNG format. Indexed by spend time and users.
	Innovatemedtec.		
	https://innovatemedtec.com		
27.	Internet statistics in India.	2015: Internet statistics in India.	Infographics. Indexed by gender, activity and area.
	Social Prachar.		
	https://socialprachar.com		
28.	Statistical report on the Internet development in China.	1997–2016: Internet statistics in China.	37 reports published annually. PDF format. Indexed by user provinces, gender, age, education, occupation, income, access, usage and app.^a^
	By China Network Information Centre CNNIC.		
	http://www.apira.org		
29.	Chinese social media statistics.	2015: Chinese social media statistics.	Infographics. PNG and BMP format. Indexed by users, usage, access, device, gender, age and app.
	Make a website hub.		
	https://makeawebsitehub.com		
30.	China internet statistics.	2009–2017: Chinese social media statistics.	Whitepapers, presentations, articles, posts. PPT, PNG, BMP and TXT format. Indexed by users, area, age, gender, education, occupation, income, access, device, activity and app.^a^
	China Internet Watch.		
	https://www.chinainternetwatch.com		
31.	Internet statistics in Vietnam.	2015–2016: Internet statistics in Vietnam.	Post published in 2016. TXT format. Indexed by users, activity, spend time, device and app.^a^
	Chabrol.		
	http://chabrol.net		
32.	Internet statistics in Russia.	2015: Internet statistics in Russia.	Article published in 2016. PNG and BMP format. Indexed by penetration, device, age and city size.^a^
	East-West digital news.		
	http://www.ewdn.com		
33.	Russian internet growth and development.	2014: Internet statistics in Russia.	Article published in 2015. JPG and BMP format. Indexed by penetration and area.^a^
	Russian Search Marketing.		
	http://russiansearchmarketing.com		
34.	Internet statistics in Malaysia.	2013 and 2015: Internet statistics in Malaysia.	Infographics published in 2016. PDF format. Indexed by usage, access, activity and device.
	Department of Statistics Malaysia.		
	https://www.dosm.gov.my		
35.	The Canadian internet.	2011–2012: Internet statistics in Canada.	Post published in 2014. PNG and BMP format. Indexed by access, area, activity, device and app.^a^
	CIRA.		
	https://cira.ca		
36.	Internet statistics in the USA.	1998–2015: Internet statistics in the USA.	Yearly updates. XLS format. Indexed by device, access, activity, age, income, employment, education, gender and race.^a^^,^^b^
	NTIA.		
	https://www.ntia.doc.gov		
37.	Global broadband penetration.	2011: Global broadband penetration.	Online article. Indexed by access, penetration and country.^a^
	Global Finance Magazine.		
	https://www.gfmag.com		
38.	Internet statistics in the Arab countries.	2013: Internet statistics in the Arab countries.	Study published in 2013. PDF format. Indexed by penetration, access, device, browsers, country, gender, age and activity.^a^
	IPSOS.		
	https://www.ipsos.com		
39.	Internet statistics.	Various Internet statistics.	Indexed by country, access, cost and speed.^a^
	Internet Monitor.		
	https://thenetmonitor.org		
40.	Global Internet Maps.	2013: Internet statistics. Internet Measurement Project.	Reports, surveys, external references to free and non-free documents.
	Internet Society.		
	http://www.internetsociety.org		
41.	Avazu.	Internet and online games statistics in Russia and Brazil.	White papers. PDF format.
	http://avazuinc.com		
42.	Social media statistics.	2016: Social media statistics.	Article published in 2016. PNG and BMP format. Indexing by app.
	UK Business Insider.		
	http://uk.businessinsider.com		
43.	Web Traffic Analytics.	2002-present: Web traffic data analytics, traffic, ranking and other information.	Data on 30 million websites. Partly free. HTML format. Complete web access statistics with paid account.
	Alexa Internet.		
	https://www.alexa.com/		

a Other data available.

b Various visualization options available.
Table 4. Internet availability: These datasets are concerned with the Internet accesses in different countries and world regions.

**Table 4:** Internet availability.

	**Data source**	**Data**	**Notes**
1.	Internet access.	2005–2015: Internet access.	Yearly updates. XLS format. Indexed by country.^a^^,^^b^
	OECD.		
	https://data.oecd.org		
2.	Internet access and use in the EU27.	2006–2008: Internet access in Europe.	Data published in 2008. PDF and DOC format. Indexed by access, activity and country.^a^
	European Commission.		
	https://ec.europa.eu		
3.	Digital economy and society statistics.	2016: Internet access for households and individuals in Europe.	Data published in 2017. XLS, PNG and BMP format. Indexed by usage, household, area, device and shopping.
	Eurostat.		
	http://ec.europa.eu		
4.	The net-children go mobile project.	2013: Internet access among European children.	Initial findings published in 2013. PDF format. Indexed by usage, age, device, activity, social media and gender.^a^
	LSE Research Online.		
	http://eprints.lse.ac.uk		
5.	Internet access statistical bulletins.	2012–2016: Internet access of households and individuals in the UK.	Yearly updates. PDF, JPG and XLS format. Indexed by device, activity, age and city.^a^
	ONS.		
	https://www.ons.gov.uk		
6.	Internet access quarterly update.	2011–2014: Internet access in the UK.	Quarterly updates; last in 2014. PDF format. Indexed by age, gender and region.^a^
	Government UK.		
	https://data.gov.uk		
7.	Internet access and use.	2013: Internet access in the UK.	Report published in 2014. PDF format. Indexed by households, usage, activity, age, city, race and gender.
	Tower Hamlets Council.		
	http://www.towerhamlets.gov.uk		
8.	Internet access datasets.	2017: 19 datasets on Internet access in Singapore.	Yearly updates. XLS format.^a^
	Singapore Gov.		
	https://data.gov.sg		
9.	Internet access among children in the USA.	1994–2013: Internet access among children in the USA.	A report. PDF format. Index by age, gender, race, income and region.
	Child Trends Org.		
	https://www.childtrends.org		
10.	Americans internet access.	2000–2014: Internet access in the USA.	Report published in 2015. PDF, SVG, PNG and BMP format. Indexed by use, age, education, income, race, area and gender.^a^^,^^b^
	Pew Research Center.		
	http://www.pewinternet.org		
11.	Computer and Internet Access in the USA.	1984–2012: Internet access in the USA.	Reports. XLS format. Indexed by age, gender, ethnicity and income.
	The U.S. Census Bureau.		
	https://www.census.gov		

a Other data available.

b Various visualization options available.
Table 5. More comprehensive datasets related to the Internet: These datasets often contain various other data in addition to the Internet data.

**Table 5:** More comprehensive datasets related to the Internet.

	**Data source**	**Data**	**Notes**
1.	Data hub.	Total 11,238 datasets with 312 about the Internet.	Different formats. In 47 langs.
	https://datahub.io		
2.	European data portal.	Total 764,778 datasets with 60,239 about the Internet.	Different formats. In 24 langs. European countries only.
	https://www.europeandataportal.eu		
3.	European Union Open Data Portal.	Total 10,850 datasets with 293 about the Internet.	Different formats. In 24 langs. EU countries.
	https://data.europa.eu		
4.	Internet live stats.	Live data about internet users, number of websites and apps.	Real-time data.
	http://www.internetlivestats.com		
5.	ITU.	Datasets about broadband, Internet use, mobile-cellular and mobile-broadband networks in the world.	Yearly updated data. Different formats. In 5 langs.
	http://www.itu.int		
6.	World Bank Group.	Total 774 million. Datasets about the Internet and other fields.	Yearly updates. Different formats. In 20 langs. Good for data on developing countries.
	http://www.worldbank.org		
7.	Statista. https://www.statista.com	Thousands of datasets about the Internet and other fields.	Yearly updates. Different formats. In 4 langs. Free and non-free access.
8.	Info Please. https://www.infoplease.com	Lots of data about the Internet and other fields in the USA and in the world.	Yearly updates. Different formats.
9.	Ofcom.	Lots of data about the Internet in the UK.	Yearly updates. Different formats.
	https://www.ofcom.org.uk		
10.	FCC	Lots of data about the Internet in the US.	Yearly updates. PDF format.
	https://www.fcc.gov		
11.	Pew Research Center. http://www.pewglobal.org	Lots of data about the Internet and other fields in the USA and in the world.	Data polling, research and analysis. Yearly updates.
12.	Internet Society. https://www.internetsociety.org	Data about the Internet.	Different formats and visualizations. In 8 langs.
13.	ONS.	Lots of data about the Internet and other fields in the UK.	Collect, analyse and publish data about the UK. Yearly updates. Different formats.
	https://www.ons.gov.uk		
14.	Stat. Counter.	Data about the Internet.	Daily updates. Different formats and visualizations. Data from 2.5 million websites. Indexed by browser, device, country, region and app.
	http://gs.statcounter.com		
15.	Our World in Data. https://ourworldindata.org	Lots of data about the Internet and many other fields.	Free data research and analysis. PNG and BMP format.
16.	UN data. http://data.un.org	Lots of data about the Internet and other fields also listing the UN organizations with the last and next update datasets.	Monthly updates. Different formats. Indexed by topic, country and time period.
17.	OECD.	Lots of data about the Internet and other fields.	Yearly updates. XLS format. Indexed by penetration, usage, price, service, speed and coverage. In 2 langs.
	http://www.oecd.org		
18.	The Global Economy.	Lots of data about the Internet and other fields.	Free and non-free data. JPG format. Other formats with free registration. Different visualizations. In 6 langs.
	http://www.theglobaleconomy.com		
19.	Slideshare.	Total 18 million presentations in 40 categories, many containing data about the Internet and other fields.	Different formats. In 5 langs.
	https://www.slideshare.net		
20.	Knoema.	Search engine for data.	Different formats. In 10 langs.
	https://knoema.com		
21.	Australian Bureau of Statistics.	Official data Australia including the Internet.	Yearly updates. XLS format.
	http://search.abs.gov.au		
22.	Internet Stats Today.	Live mobile usage statistics.	Live updates. Indexed by activity, device and app.
	http://internetstatstoday.com/		
23.	Apira.	The Internet developments in countries in the Asia-Pacific region.	Survey reports and Internet data in different countries.
	http://www.apira.org		
24.	ABI research	Research on technology markets.	Quarterly updates. Data and reports on the Internet, markets and technology. Registration required. Data access not free.^a^
	https://www.abiresearch.com/		
25.	Eurostat	Lots of data about the Internet and other things in Europe.	Data about the Internet in Europe. Indexed by user, access, activity and security. Many formats.
	http://ec.europa.eu		
26.	Pingdom	Data and analysis on the Internet.	Lots of data about the Internet and other fields.
	http://royal.pingdom.com		
27.	Strategy Analytics	Data on the Internet and other fields.	Lots of data and reports, but registration required.
	https://www.strategyanalytics.com		
28.	PWC	Investment data in the USA including the Internet.	Quarterly update. SVG, PNG and BMP format. Indexed by sector, region, state.
	https://www.pwc.com		
29.	UNCTAD statistics	United Nations Conference on Trade and Development. Data on ICT and Internet usage and economics.	Yearly update. IVT, XLS and CSV format. In 2 langs.
	http://unctadstat.unctad.org		
30.	Statistics Canada	Data on the Internet and other things in Canada.	Data about the Internet and other statistics.
	http://www.statcan.gc.ca		

a Other data available.

## 2 Experimental design, materials and methods

**Fig. 1:**
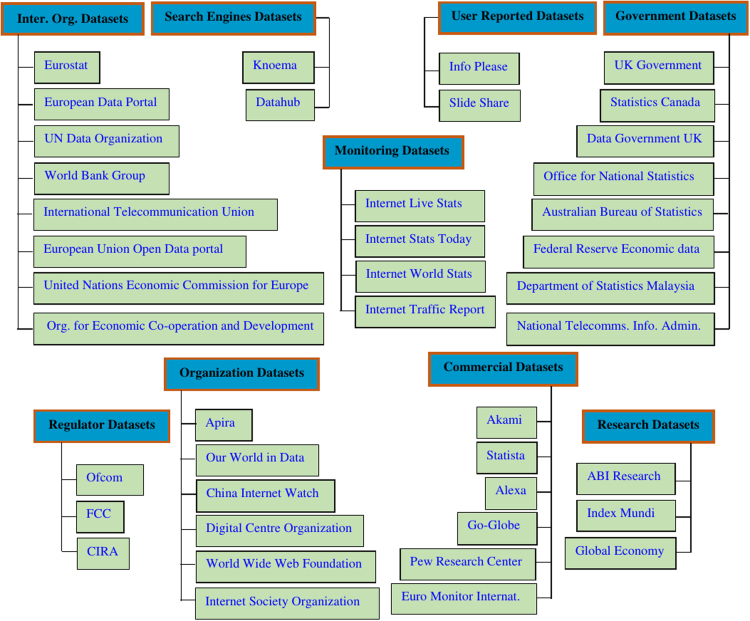
The most comprehensive datasets on the Internet usage and other Internet statistics.

The most important data sources on the Internet usage containing the most comprehensive datasets are summarized in Fig. 1. The following two main strategies were used to find the relevant datasets. First, the World Wide Web (WWW) was searched using various combinations of keywords to find about 80% of the collected datasets. The keywords considered combined the word `Internet’ with one or more of the following search terms: traffic, database, data, access, usage, users, and statistics. The keyword search can be further modified, for example, by specifying the country or a region. The second strategy for finding the relevant datasets is to first identify the institutions which are known to be reporting various Internet related statistics, and then examine their websites. Overall, over 400 candidate websites were explored to select the relevant datasets. It is not surprising that often, but fortunately, not always, the high quality datasets (i.e., those which are reliable and regularly kept up to date) are only available to the paying subscribers. However, these pay-for-access datasets have been excluded from the survey. The main attributes of interest that were checked for when screening the datasets are: whether the data are provided for free, who is maintaining the dataset, how often the data are updated, in what format the data are available, and especially, what data are specifically provided and how they are indexed or categorized. Moreover, many international institutions have a policy to publish datasets with description in multiple languages. The total volume of data in each dataset varies significantly among the data providers including the time coverage and the availability of historical data. Most data seem to be normally accessible in smaller chunks, usually organized around a specific reporting purpose or issue.

References [1], [2], [3], [4], [5] are recommended for performing longer-term traffic and applications forecasting in the Internet. The Internet measurement procedures are described in [6], [7], [8], [9] whereas focus on measuring the digital economy can be found in [10], [11], [12]. Digitalization of the economy is explained in [13] while many technical and business aspects of digitalization of the Internet services are studied in [14]. The value of open access data and the associated data regulation are discussed in [15] and [16].

## Funding sources

This work is part of a Ph.D. research work of Murooj Nadhom who received the full scholarship from the Iraqi Government. The grant is provided by the Iraqi Ministry of Higher Education and Scientific Research. The grant reference number is 1873.
