# Supplementary material for: Survey of public data sources on the Internet usage and other Internet statistics
Source: Data Brief. 2018 May 9;18:1914–29. doi: 10.1016/j.dib.2018.04.107 (PMC5998297; doi:10.1016/j.dib.2018.04.107)
Supplement: Supplementary file 1 — Transparency document [file mmc1.doc]

6 April 2018

**Survey of public data sources on the Internet usage and other Internet statistics**

Murooj Nadhom and Pavel Loskot

Declarations of interest: none
